# Normative values for esophageal functional lumen imaging probe measurements: A meta‐analysis

**DOI:** 10.1111/nmo.14419

**Published:** 2022-06-05

**Authors:** Albert J. Bredenoord, Francesca Rancati, Haiying Lin, Naama Schwartz, Mirit Argov

**Affiliations:** ^1^ Department of Gastroenterology and Hepatology Amsterdam UMC Amsterdam The Netherlands; ^2^ Medtronic Minneapolis Minnesota USA

**Keywords:** achalasia, catheter, dysphagia, esophagus, manometry, reflux disease

## Abstract

**Background:**

The functional lumen imaging probe (Endoflip™) is increasingly used for evaluation of patients with esophageal symptoms. To improve the interpretation of Endoflip™ in clinical practice, normative values with appropriate cut‐off values are required.

**Methods:**

Original clinical studies describing Endoflip™ use for measurements of esophageal motility in healthy adults were considered. Meta‐analysis was performed based on published values.

**Results:**

A total of 17 articles were included in the systematic review, 15 of which were included in the meta‐analysis, representing 154 unique subjects. At 40 ml distention, the 5th–95th and 10th–90th percentiles for esophagogastric junction distensibility index (EGJ‐DI) were 1.96–10.95 mm^2^/mmHg and 2.36–8.95 mm^2^/mmHg, respectively. An EGJ‐DI below 2 mm^2^/mmHg was found in 5.4%, and below 3 mm^2^/mmHg in 20.1% of healthy subjects. At 50 ml distention, the 5th–95th and 10th–90th percentiles for EGJ‐DI are 2.86–10.66 mm^2^/mmHg and 3.28–9.12 mm^2^/mmHg, respectively (below 2 mm^2^/mmHg: 0.6%, 3 mm^2^/mmHg: 6.3%). The 5th–95th and 10th–90th percentiles for EGJ‐DI at 60 ml distention were 3.06–8.14 mm^2^/mmHg and 3.33–7.18 mm^2^/mmHg, respectively (below 2 mm^2^/mmHg: 0.0%, 3 mm^2^/mmHg: 7%). A clear cut‐off for lower values was identified while a large spread in values was observed for upper limits of normal for EGJ‐DI for all filling volumes.

**Conclusions:**

Given these observations, we recommend using a cut‐off of 2 mm^2^/mmHg for clinical practice, values below can be considered abnormal. Given that 5.4% of the healthy subjects will have an EGJ‐DI below 2 mm^2^/mmHg at 40 ml, we recommend using the 50 and 60 ml distention volumes. The clinical use of an upper limit for normality of EGJ‐DI seems questionable.

## Graphical abstract

A meta‐analysis of clinical studies describing Endoflip™ use for measurements of esophagogastric junction distensibility index (EGJ‐DI) in healthy subjects reveals that only 5.4% of subjects have an EGJ‐DI of 2 mm^2^/mmHg or lower, values below it can be considered abnormal. The clinical use of an upper limit for normality of EGJ‐DI seems questionable.

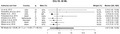

**Key points**

- At a distention volume of 40 ml, 95% of normal subjects will have a EGJ‐DI above 2 mm^2^/mmHg, values below are considered abnormal.
- At 60 ml distention volume, EGJ‐DI values below 3 mm^2^/mmHg are considered abnormal.
- The clinical use of an upper limit for normality of EGJ‐DI seems questionable.

## 1 INTRODUCTION

The functional lumen imaging probe (Endoflip™) is a device consisting of a catheter with multiple pairs of impedance electrodes and pressure sensors, surrounded by a fluid‐inflatable balloon and a mechanical pump, controlling fluid‐inflation at known speed and volume.^1^ The device allows interrogation of the tubular organs and sphincters such as esophagus, esophagogastric junction (EGJ), pyloric sphincter and anal sphincter and provides information about distensibility and cross‐sectional area (CSA) of these organs at known pressures. Two different Endoflip™ catheters are available, one shorter catheter (EF‐325N: 8 cm catheter with 16 sensors spaced 0.5 cm apart) designed to examine the EGJ and a longer catheter (EF‐322N: 16 cm catheter with 16 sensors spaced 1 cm apart) designed to examine both secondary peristaltic patterns of the esophageal body as well as examination of the EGJ. Catheter insertion at the start of the Endoflip™ procedure is recommended to be performed in most cases under endoscopic visualization and the endoscope should be removed prior to initiating the Endoflip™ protocol. Endoflip™ may also be used as a stand‐alone test when inconclusive results from other diagnostic tests require a confirmative test.^2^, ^3^, ^4^

Endoflip™ is increasingly used in clinical esophagology to evaluate patients with dysphagia, chest pain and heartburn, it is particularly popular in evaluation of patients with suspected or treated achalasia. Using Endoflip™, it has been shown that distensibility is markedly reduced in patients with achalasia compared to controls and an increase of distensibility after achalasia treatment is associated with improvement of symptoms.^4^, ^5^, ^6^ Furthermore, Endoflip™ helps to identify achalasia patients in whom esophageal manometry is inconclusive if the integrated relation pressure (IRP) is within the normal range. In the recently released Chicago Classification version 4.0, Endoflip™ is recommended in patients with so‐called EGJ outflow obstruction (EGJOO), who have preserved peristaltic contractions but an elevated IRP, as it can help to distinguish true EGJOO from artefactual EGJOO.^7^ In contrast, studies show that EGJ distensibility is increased in patients with GERD, which makes sense from a pathophysiological perspective as these patients often have a weakened anti‐reflux barrier.^8^

However, to improve the interpretation of Endoflip™ in clinical practice, normative values with appropriate cut‐off values are required.^2^ Normative data are already available from small case series, where the data from healthy subjects were used to compare with those with certain disease conditions, but the scattered data does not provide a normative range that can be conveniently used in clinical practice. Therefore, our objective was to review the current literature and provide an overview of all measurements performed in healthy subjects and perform a meta‐analysis on the pooled data to provide normative values for this diagnostic test. This manuscript is intended to inform and facilitate proper on‐label use of Endoflip™ technology in symptomatic patients.

## 2 MATERIALS AND METHODS

### 2.1 Systematic review search

**FIGURE 1:**
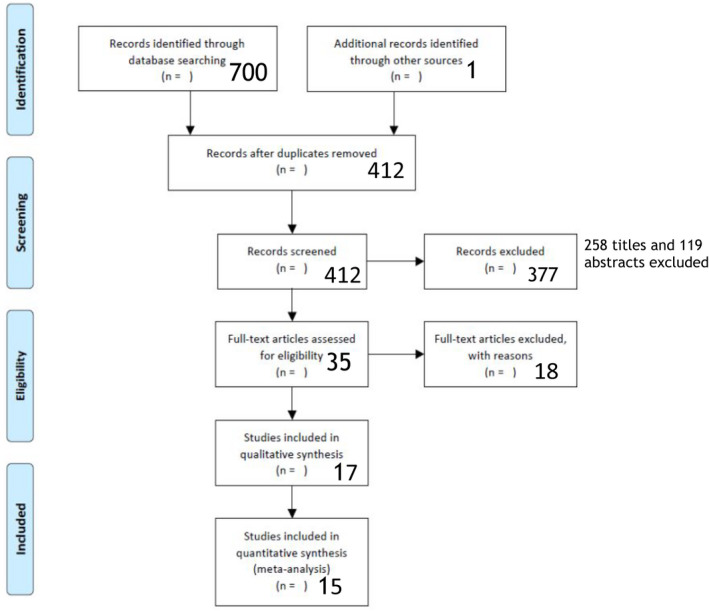
PRISMA flow diagram. PRISMA flow diagram depicting the systematic review process

A comprehensive review of the medical literature was conducted for studies that reported Endoflip™ system measurements on healthy, asymptomatic subjects. Relevant, full‐text, original publications were identified using MEDLINE, EMBASE, Journals@Ovid, and through independent search. Prespecified medical terms were used for the search and are reported in Table S1. To ensure data presented in this review was up to date at the time of publication, an additional search of abstracts published between January 2017 and June 2020 was conducted to evaluate whether any additional publications were expected in the near future. No abstracts were identified that contained novel data. The review of articles, analyses, and inclusion criteria were based on PRISMA recommendations^9^ and the process is summarized in Figure 1.

All clinical studies that included use of the Endoflip™ system in healthy adults from January 2000 to December 2020 were considered. Only publications that included original data and were available in English were included. Abstracts, conferences, reviews, case reports, guidelines, and consensus documents were excluded.

The titles and abstracts of all retrieved articles were screened by two independent reviewers (M.A. and F.R. or H.C.), according to the prespecified search terms. In the event of any disagreements or concerns, a third reviewer was consulted (A.J.B.). If there was any suspicion of cohort overlap between studies, the most comprehensive publication was used for the respective Endoflip™ system parameter. Studies were included if they reported the use of Endoflip™ system to assess distal esophageal motility in asymptomatic adult subjects, aged 18 years and over. Studies were excluded if they (1) did not report original data (reviews, guidelines, expert consensus document), (2) did not report any information on Endoflip™ system measurements, (3) did not report Endoflip™ system measurements in asymptomatic subjects, (4) did not report any information on Endoflip™ system EGJ or esophageal body measurements, (5) included less than four subjects, or (6) reported pediatric population measurements.

### 2.2 Data collection

The following information was extracted from included publications: (1) author; (2) journal; (3) year of publication; (4) type of publication (manuscript or abstract); (5) country(ies) of publication; (6) single or multicenter study; (7) study design (prospective or retrospective); (8) objective; (9) sample size; (10) patient demographics (mean age and standard deviation, sex); (11) catheter type; (12) sedation type; (13) sedation agent; (14) placement; (15) pressure zero; (16) balloon fill volumes; (17) EGJ distensibility index (EGJ‐DI); (18) distal esophageal body diameter; (19) maximum diameter; (20) RACs Rate (Number of Antegrade Contractions in 60 seconds); (21) RACs trigger volume; (22) RACs stop volume; (23) contractile pattern (number of antegrade contractions, number of retrograde contractions, contractility without RACs/ RRCs, absent contractility). Primary outcomes of interest were EGJ‐DI, intrabag pressure, CSA, and maximum diameter.

Quality assessment of each study was completed using the Newcastle‐Ottawa Scale (NOS).^10^ This checklist scores for three parameters of quality: (1) subject selection, (2) comparability of groups where appropriate, and (3) exposure (case control) or outcome (cohort) of interest. As only the data of healthy controls were used in this meta‐analysis, the second parameter of the NOS (comparability of groups) was not employed when assessing the quality of the studies. Possible scores range from 0 to 4 stars for subject selection, 0 to 2 stars for comparability and 0 to 3 stars for exposure/outcome.

### 2.3 Statistical methods

Analyses were performed using R (Version 4.0.3). Endoflip™ system studies of normative data are generally very small, with data being highly skewed and not normally distributed. Furthermore, study outcomes were not consistently reported across included studies. Some reported data as median (IQR), median (range), or mean (SD). Statistical summaries were calculated using percentiles, quantile estimation, and distribution fitting, as appropriate. Imputation for missing data and analysis of variance were also employed as needed. Meta‐analysis was performed based on median values along with IQR [25th, 75th quantiles], [5th, 95th] or [10th, 90th] percentiles, as appropriate. Mixed‐effects models with random effects were used to account for heterogeneity across studies. Heterogeneity between studies was assessed by Cochran’s *Q* test^11^ and Higgins & Thompson's *I* ^2^.^12^ For each outcome, descriptive statistics are provided, along with [5th, 95th] and [10th, 90th] percentiles. Forest plots were used to summarize individual study, and overall results. Funnel plots were used to assess potential publication biases. Significance was set at α = 0.05. No sensitivity, subgroups analysis, or meta‐regression were performed due to the small‐scale of the included studies.

## 3 RESULTS

### 3.1 Systematic review results

A total of 700 articles were identified through database search and one article through independent search. Figure 1 provides a detailed summary of the stepwise article selection process. After the removal of 288 duplicate records, 258 publications were excluded based on title, and 119 articles were excluded based on abstracts. A total of 35 full‐text articles were assessed, of which 20 were excluded, leaving a total of 17 articles^5^, ^6^, ^13^, ^14^, ^15^, ^16^, ^17^, ^18^, ^19^, ^20^, ^21^, ^22^, ^23^, ^24^, ^25^, ^26^, ^27^ included in the systematic review, 15 of which were included in the meta‐analysis.^5^, ^6^, ^13^, ^14^, ^15^, ^16^, ^18^, ^19^, ^20^, ^21^, ^22^, ^24^, ^25^, ^26^, ^27^ The findings presented by Lin et al.^18^ and Carlson et al.^14^ came from the same dataset. The publications by Kwiatek et al.^20^ and Pandolfino et al.^6^ presented unique information and were derived from the same dataset. In addition to 12 unique subjects, the dataset used by Rooney et al.^27^ included data previously presented Lin et al.,^18^ and Carlson et al.^14^ To prevent duplications, data from subjects that were included in multiple publications was only considered once in the meta‐analysis. A total of 154 subjects from 15 studies were included, refer to Table 1 for details.

**TABLE 1:** Characteristics of included studies

Author, Year	Study type	Country	Sample size of control (healthy) group	% Female	Age	Catheter type	Measures collected (distension volume, ml)	NOS score
Kwiatek et al., 2010^20^	Case control	USA	20	80.0	Range: 18–42 years	EF‐325N	Intrabag pressure (20, 30, 40) CSA (20, 30, 40) EGJ‐DI (20, 30) Maximum diameter (20, 30, 40)	6
Kwiatek et al., 2011^19^	Case control	USA	15	60.0	Range: 21–68 years	EF‐325N	Intrabag pressure (20, 30) CSA (20, 30) EGJ‐DI (20, 30) Maximum diameter (20, 30)	6
Rohof et al., 2012^5^	Cohort	The Netherlands	15	46.7	Mean ± SEM: 40 ± 4.1 years	EF‐325N	EGJ‐DI (50)	6
Lin et al., 2013^18^	Cohort	USA	10	60.0	Range: 21–49 years	EF‐322N	EGJ‐DI (30, 40, 60)	5
Rieder et al., 2013^25^	Case control	USA	4	Not reported	Not reported	EF‐325N	Intrabag pressure (30, 40) CSA (30, 40) EGJ‐DI (30, 40) Maximum diameter (30, 40)	4
Pandolfino et al., 2013^6^	Case control	USA	20	80	Range: 18–42 years	EF‐325N	EGJ‐DI (20, 30, 40)	5
Tucker et al., 2013^17^	Case control	UK	21	76.2	Range: 22–46 years	EF‐325N		6
Fukazawa et al., 2014^22^	Cohort	Japan	9	0	Range: 21–52 years	EF‐325N	Intrabag pressure (20, 40, 50) CSA (20, 40, 50) EGJ‐DI (20, 40, 50) Maximum diameter (20, 40, 50)	
Carlson et al., 2015^14^	Case control	USA	10	60	Range: 20–49 years	EF‐322N	EGJ‐DI (50)	7
Smeets et al., 2015^26^	Case control	The Netherlands	15	66.7	Range: 19–50 years	EF‐325N	Intrabag pressure (30, 40, 50) CSA (30, 40, 50) EGJ‐DI (30, 40, 50) Maximum diameter (30, 40, 50)	5
Lottrup et al., 2016^21^	Cohort	Denmark	10	40	Mean ± SD: 52.6 ± 9.3 years	EF‐325N	Intrabag pressure (20, 30, 40, 50) CSA (20,30, 40, 50) EGJ‐DI (20, 30, 40, 50) Maximum diameter (20, 30, 40, 50)	5
Mikami et al., 2016^15^	Cohort	Japan	9	0	Mean ± SEM: 25.4 ± 3.1 years	EF‐325N	Intrabag pressure (20, 30, 40) CSA (20, 30, 40) EGJ‐DI (20, 30, 40 Maximum diameter (20, 30, 40)	6
Fynne et al., 2017^23^	Case control	Denmark	11	90.9	Range: 40–68 years	EF‐325N		8
Liao et al., 2018^24^	Cohort	Denmark	6	Not reported	Mean ± SEM: 53.8 ± 10.3 years	EF‐325N	Intrabag pressure (20, 30, 40)	6
Mikami et al., 2018^16^	Cohort	Japan	9	33.3	Range: 43–63 years	EF‐325N	Intrabag pressure (20, 30, 40) CSA (20, 30, 40) EGJ‐DI (20, 30, 40) Maximum diameter (20, 30, 40)	6
Carlson et al., 2019^13^	Cohort	USA	20	70.0	Range: 23–44 years	EF‐322N	Intrabag pressure (60) CSA (60) EGJ‐DI (60) Maximum diameter (60)	6
Rooney et al., 2020^27^	Case control	USA	42	69.0	Mean ± SD: 31 ± 6 years	EF‐322N	Intrabag pressure (60)	6

*Note*: The sample sizes presented in this table reflect only the control (healthy) group in each respective study and does not include symptomatic subjects.

Abbreviations: CSA, cross‐sectional area; EGJ‐DI, esophagogastric junction distensibility.

### 3.2 Demographics and characteristics of included studies

A total of 15 publications^5^, ^6^, ^13^, ^14^, ^15^, ^16^, ^18^, ^19^, ^20^, ^21^, ^22^, ^24^, ^25^, ^26^, ^27^ were included in the meta‐analysis, which was comprised of 11 unique data sets^5^, ^13^, ^15^, ^16^, ^19^, ^21^, ^22^, ^24^, ^25^, ^26^, ^27^ and represented a total of 154 unique subjects (76 females, 68 males, 10 gender not reported^24^, ^25^, ^27^). A summary of included studies is presented in Table 1. Eight studies were completed in the United States,^6^, ^13^, ^14^, ^18^, ^19^, ^20^, ^25^, ^27^ three studies were completed in Denmark,^21^, ^23^, ^24^ three studies were completed in Japan,^15^, ^16^, ^22^ two studies were completed in The Netherlands,^5^, ^26^ and one study was completed in the United Kingdom.^17^ Sample size ranged from 4 to 42 subjects (median = 10) and female representation ranged from 0%–91% (median = 46.7%) across trials. All studies scored 2–4 out of 4 possible stars for subject selection, 0–1 out of possible 2 stars for comparability and 2–3 out of 3 stars for outcome assessment, studies thus performed poorly on comparability but fair to good on subject selection and exposure/outcome assessment. As the aim of our analysis was not centered on comparability between controls and patients, all studies were determined to be of sufficient quality for inclusion in the analysis. Exact definition and criteria used to determine healthy status differed by study and was dependent on condition of interest, though presenting as asymptomatic was a common criterion among all studies that reported description of the healthy sample. Three studies^13^, ^17^, ^23^ performed study procedures under conscious sedation (midazolam and fentanyl,^13^ midazolam only,^23^ or midazolam and pethidine^17^), six studies^6^, ^14^, ^18^, ^19^, ^20^, ^21^ performed study procedures under moderate sedation (midazolam and fentanyl), two studies^5^, ^15^ used local anesthesia (lidocaine), four studies^22^, ^24^, ^25^, ^26^ used no sedation, and two studies^16^, ^27^ did not report sedation information. Two studies^13^, ^27^ used the EF‐322 catheter, two studies^14^, ^18^ used the EF‐322N catheter, and 13 studies^5^, ^6^, ^15^, ^16^, ^17^, ^19^, ^20^, ^21^, ^22^, ^23^, ^24^, ^25^, ^26^ used the EF‐325N catheter. Catheters had transoral placement for all studies that reported placement. Information on RACs Rate, RACs trigger volume, RACs stop volume; and contractile pattern was only available for one publication,^13^ so no further analysis was performed on these variables.

### 3.3 Outcomes

Thirteen studies^5^, ^6^, ^13^, ^14^, ^15^, ^16^, ^18^, ^19^, ^20^, ^21^, ^22^, ^25^, ^26^ were included in the meta‐analysis for EGJ‐DI for at least one distension volume level (20–60 ml), 10 studies^15^, ^16^, ^19^, ^20^, ^21^, ^22^, ^24^, ^25^, ^26^, ^27^ were included in the meta‐analysis for intrabag pressure for at least one distension volume measure (20–60 ml), nine studies^13^, ^15^, ^16^, ^19^, ^20^, ^21^, ^22^, ^25^, ^26^ were included in the meta‐analysis for CSA for at least one distension volume measure (20–60 ml), and nine studies^13^, ^15^, ^16^, ^19^, ^20^, ^21^, ^22^, ^25^, ^26^ were included in the meta‐analysis for maximum diameter for at least one distension volume measure (20–60 ml). Included study catheters were the EF‐322N and EF‐325N. EGJ‐DI measurement was based on combined data using the EF‐322N and EF‐325N catheters. Intrabag pressure, CSA, and maximum diameter measurements at 20–50 ml were analyzed with data from the EF‐325N catheter alone. At 60 ml, only the EF‐322N catheter data was available. Real and notable differences in methodology between studies, along with small sample sizes, contributed to a substantial amount of heterogeneity across studies, prompting use of random effects meta‐analysis models for all analyses.

### 3.4 EGJ‐DI

#### 3.4.1 20 ml

**FIGURE 2:**
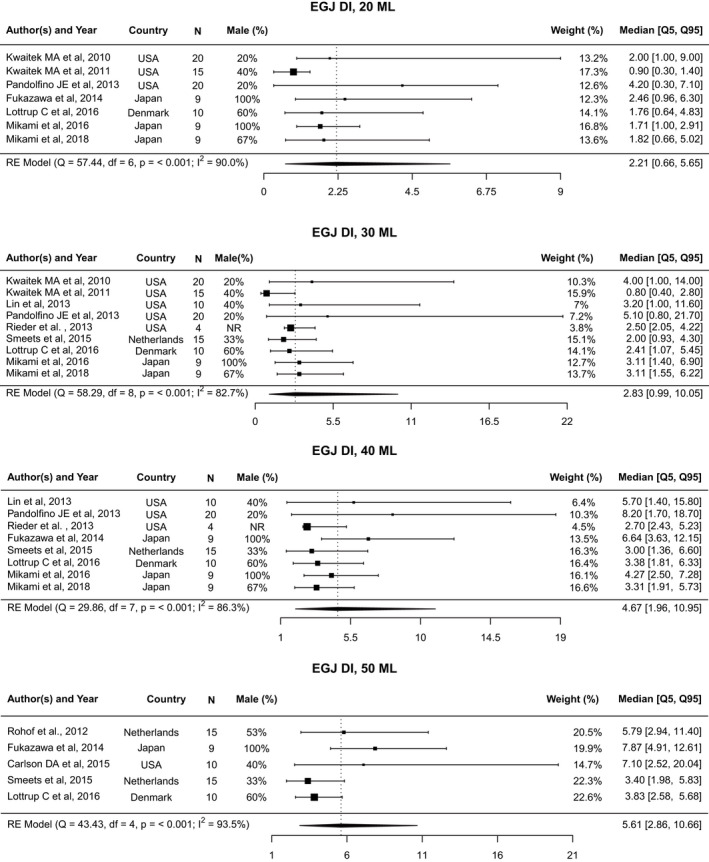
Forest plot of EGJ‐DI. Forest plot of esophagogastric junction distensibility index (EGJ‐DI) at 20, 30, 40, and 50 ml distention volumes, represented as median value and [5th, 95th] percentiles

Data from seven studies^6^, ^15^, ^16^, ^19^, ^20^, ^21^, ^22^ were included in the analysis of EGJ‐DI at a distention volume of 20 ml (Figure 2). The pooled median was 2.21 mm^2^/mmHg (5th–95th percentile: 0.66–5.65 mm^2^/mmHg; 10th–90th percentile: 0.85–4.45 mm^2^/mmHg; Figure S1), with significant heterogeneity observed across studies (*I* ^2^ = 90.0%; Cochran’s *Q* = 57.4, *p* < 0.001). The funnel plot appears asymmetric, with two studies^6^, ^19^ outside the triangular 95% confidence region (Figure S5A).

#### 3.4.2 30 ml

The pooled median for EGJ‐DI at a distention volume of 30 ml was 2.83 mm^2^/mmHg (5th–95th percentile: 0.99–10.05 mm^2^/mmHg; 10th–90th percentile: 1.24–7.52 mm^2^/mmHg), using data from nine studies.^6^, ^15^, ^16^, ^18^, ^19^, ^20^, ^21^, ^25^, ^26^ Notable heterogeneity was detected (*I* ^2^ = 82.7%; Cochran’s *Q* = 58.3, *p* < 0.001). Furthermore, the funnel plot appears asymmetric, with two studies^19^, ^26^ appearing as outliers (Figure S5B).

#### 3.4.3 40 ml

Analysis of EGJ‐DI at 40 ml distention volume used data from eight studies,^6^, ^15^, ^16^, ^18^, ^21^, ^22^, ^25^, ^26^ with a pooled median of 4.67 mm^2^/mmHg (5th–95th percentile: 1.96–10.95 mm^2^/mmHg; 10th–90th percentile: 2.36–8.95 mm^2^/mmHg). Here again, we see significant heterogeneity across studies (*I* ^2^ = 86.3%; Cochran’s *Q* = 29.9, *p* < 0.001) and the funnel plot appears asymmetric (Figure S5C). The percent of measurements for EGJ‐DI below 2 mm^2^/mmHg was 5.4%, while the percent of measurements for EGJ‐DI below 3 mm^2^/mmHg was 20.1% (Table 2)

**TABLE 2:** Parameter estimates for EGJ‐DI at 40, 50 and 60 ml

Volume	Parameter estimate for EGJ‐DI
40 ml	2.5% quantile of EGJ‐DI measurements: Q2.5 = 1.91 % of measurements below EGJ‐DI 2 mm^2^/mmHg = 5.4% % of measurements below EGJ‐DI 3 mm^2^/mmHg = 20.1%
50 ml	2.5% quantile of EGJ‐DI measurements: Q2.5 = 2.54 % of measurements below EGJ‐DI 2 mm^2^/mmHg = 0.6% % of measurements below EGJ‐DI 3 mm^2^/mmHg = 6.3%
60 ml (Rooney 2020)	2.5% quantile of EGJ‐DI measurements: Q2.5 = 3.13 % of measurements below EGJ‐DI 2 mm^2^/mmHg = 0% % of measurements below EGJ‐DI 3 mm^2^/mmHg = 7%

*Note*: Use 2 & 3 mm^2^/mmHg as a cut‐off to evaluate how many subjects actually have measurements below these values.

Abbreviation: EGJ‐DI, esophagogastric junction distensibility.

#### 3.4.4 50 ml

For EGJ‐DI at a distention volume of 50 ml, five studies^5^, ^14^, ^21^, ^22^, ^26^ were included in the analysis. The pooled median for EGJ‐DI at this volume was 5.61 mm^2^/mmHg (5th–95th percentile: 2.86–10.66 mm^2^/mmHg; 10th–90th percentile: 3.28–9.12 mm^2^/mmHg). Heterogeneity of studies was present (*I* ^2^ = 93.5%; Cochran’s *Q* = 43.4, *p* < 0.001) and an asymmetrical funnel plot, where three of the five studies^21^, ^22^, ^26^ fell outside the triangular 95% confidence region, was observed (Figure S5D). The percent of measurements for EGJ‐DI below 2 mm^2^/mmHg was 0.6% and 6.3% of measurements were below 3 mm^2^/mmHg (Table 2).

#### 3.4.5 60 ml

Data on EGJ‐DI at 60 ml distention were available from one study.^27^ The median was 5.60 mm^2^/mmHg (5th–95th percentile: 3.06–8.14 mm^2^/mmHg; 10th–90th percentile: 3.33–7.18 mm^2^/mmHg). The percent of measurements for EGJ‐DI below 2 mm^2^/mmHg was 0.0% and 7% of measurements were below 3 mm^2^/mmHg (Table 2).

### 3.5 Intrabag pressure

#### 3.5.1 20 ml

**FIGURE 3:**
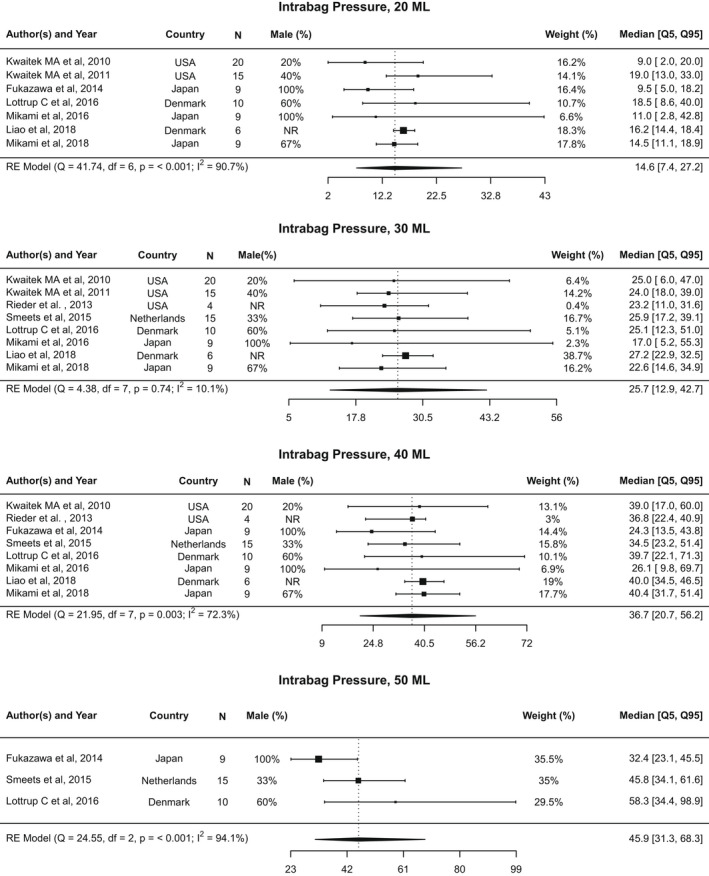
Forest plots of intrabag pressure. Forest plot of intrabag pressure at 20, 30, 40, and 50 ml distention volumes, represented as median value and [5th, 95th] percentiles

There were seven studies^15^, ^16^, ^19^, ^20^, ^21^, ^22^, ^24^ with data available for intrabag pressure at a distension volume of 20 ml. As seen in Figure 3, the pooled median intrabag pressure for asymptomatic individuals at this volume was 14.6 mmHg (5th–95th percentile: 7.4–27.2 mmHg; 10th–90th percentile: 8.2–22.6 mmHg; Figure S2). A high amount of heterogeneity was observed across studies (*I* ^2^ = 90.7%; Cochran’s *Q* = 41.7, *p* < 0.001). Three studies^20^, ^22^, ^24^ fell outside the triangular 95% confidence region on the funnel plot (Figure S6A).

#### 3.5.2 30 ml

Data from eight studies^15^, ^16^, ^19^, ^20^, ^21^, ^24^, ^25^, ^26^ were included in the analysis of intrabag pressure at a volume of 30 ml, with a pooled median of 25.7 mmHg (5th–95th percentile: 12.9–42.7 mmHg; 10th–90th percentile: 14.4–36.5 mmHg). No heterogeneity was observed across studies (*I* ^2^ = 10.1%; Cochran’s *Q* = 4.4, *p* = 0.74) and a symmetric shape appears on the funnel plot (Figure S6B).

#### 3.5.3 40 ml

The pooled median intrabag pressure for asymptomatic individuals at 40 ml distension volume was 36.7 mmHg (5th–95th percentile: 20.7–56.2 mmHg; 10th–90th percentile: 22.9–49.9 mmHg), using data from eight studies.^15^, ^16^, ^20^, ^21^, ^22^, ^24^, ^25^, ^26^ Heterogeneity was observed across studies (*I* ^2^ = 72.3%; Cochran’s *Q* = 22.0, *p* = 0.003). The funnel plot appears symmetric, although two studies^22^, ^24^ fell outside the triangular 95% confidence region (Figure S6C).

#### 3.5.4 50 ml

Three studies^21^, ^22^, ^26^ were included in the analysis of intrabag pressure at a volume of 50 ml, with a pooled median of 45.9 mmHg (5th–95th percentile: 31.3–68.3 mmHg; 10th–90th percentile: 34.0–62.5 mmHg). As with previous measures, heterogeneity was observed across studies (*I* ^2^ = 94.1%; Cochran’s *Q* = 24.6, *p* < 0.001). Two of the three studies^21^, ^22^ fell outside the triangular 95% confidence region on the funnel plot (Figure S6D).

#### 3.5.5 60 ml

Data from one study^27^ were available for intrabag pressure at a volume of 60 ml. The median intrabag pressure for asymptomatic individuals at this volume was 44.0 mmHg (5th–95th percentile: 31.8–57.7 mmHg; 10th–90th percentile: 33.1–52.5 mmHg).

### 3.6 Cross‐sectional area

#### 3.6.1 20 ml

**FIGURE 4:**
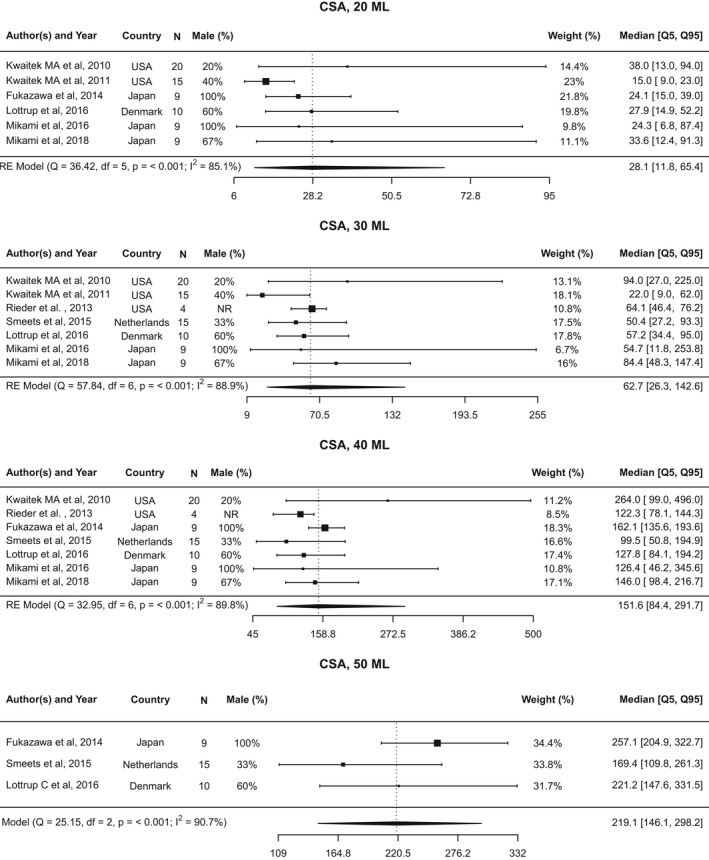
Forest plot of CSA. Forest plot of cross‐sectional area (CSA) at 20, 30, 40, and 50 ml distention volumes, represented as median value and [5th, 95th] percentiles

Data from six studies^15^, ^16^, ^19^, ^20^, ^21^, ^22^ were used to assess CSA at 20 ml volume (Figure 4). The overall median was 28.1 mm^2^ (5th–95th percentile: 11.8–65.4 mm^2^; 10th–90th percentile: 13.9–52.5 mm^2^; Figure S3). There was significant heterogeneity observed across studies (*I* ^2^ = 85.1%; Cochran’s *Q* = 36.4, *p* < 0.001). An asymmetric shape was observed in the funnel plot (Figure S7A), with one study^19^ appearing as an outlier.

#### 3.6.2 30 ml

Data for CSA at 30 ml volume were available from seven studies,^15^, ^16^, ^19^, ^20^, ^21^, ^25^, ^26^ with a pooled median of 62.7 mm^2^ (5th–95th percentile: 26.3–142.6 mm^2^; 10th–90th percentile: 30.8–114.3 mm^2^). Again, significant heterogeneity was observed across studies (*I* ^2^ = 88.9%; Cochran’s *Q* = 57.8, *p* < 0.001). An asymmetric shape was observed in the funnel plot, with two studies^16^, ^19^ falling outside the triangular 95% confidence region (Figure S7B).

#### 3.6.3 40 ml

The pooled median CSA for asymptomatic individuals at 40 ml volume was 151.6 mm^2^ (5th–95th percentile: 84.4–291.7 mm^2^; 10th–90th percentile: 95.0–249.4 mm^2^), using data from seven studies.^15^, ^16^, ^20^, ^21^, ^22^, ^25^, ^26^ Heterogeneity was observed across studies (*I* ^2^ = 89.8%; Cochran’s *Q* = 33.0, *p* < 0.001). A symmetric shape was observed on the funnel plot, with two outliers noted^20^, ^26^ (Figure S7C).

#### 3.6.4 50 ml

Analysis of CSA at 50 ml volume included data from three studies.^21^, ^22^, ^26^ The pooled median was 219.1 mm^2^ (5th–95th percentile: 146.1–298.2 mm^2^; 10th–90th percentile: 157.8–275.1 mm^2^). Significant heterogeneity was observed across studies (*I* ^2^ = 90.7%; Cochran’s *Q* = 25.2, *p* < 0.001). Two of the three studies^22^, ^26^ fell outside the triangular 95% confidence region on the funnel plot, reflective of the high degree of heterogeneity in the studies (Figure S7D).

#### 3.6.5 60 ml

Only one study^13^ included data on CSA at 60 ml, with a reported median of 274.0 mm^2^ (5th–95th percentile: 172.7–434.7 mm^2^; 10th–90th percentile: 191.2–392.6 mm^2^).

### 3.7 Maximum diameter

#### 3.7.1 20 ml

**FIGURE 5:**
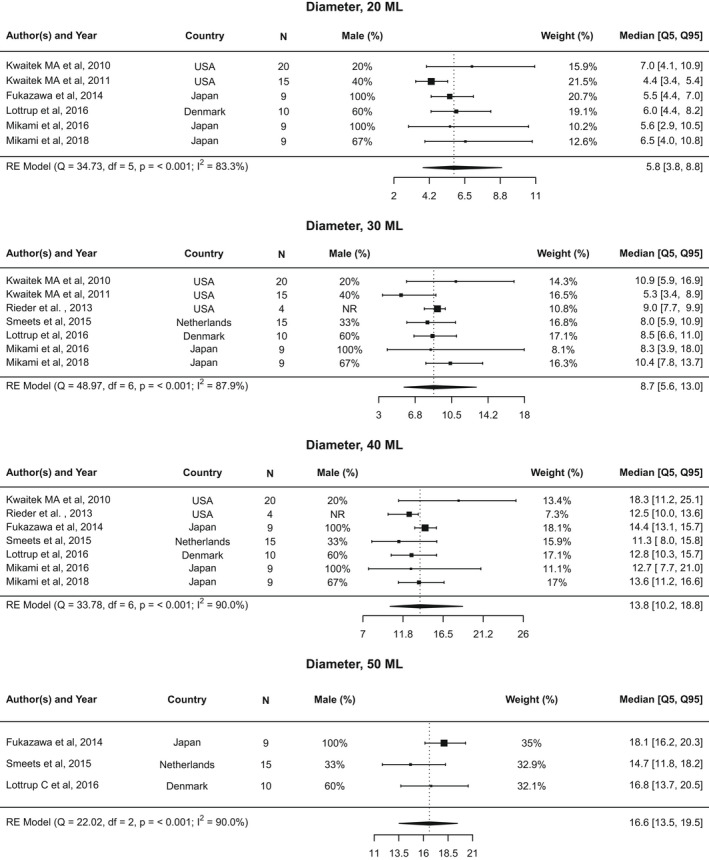
Forest plot of diameter. Forest plot of diameter at 20, 30, 40, and 50 ml distention volumes, represented as median value and [5th, 95th] percentiles

Data on maximum diameter measures at 20 ml distention volume were included from six studies^15^, ^16^, ^19^, ^20^, ^21^, ^22^ (Figure 5). The pooled median was 5.8 mm (5th–95th percentile: 3.8–8.8 mm; 10th–90th percentile: 4.2–8.0 mm; Figure S4). Notable heterogeneity was observed across studies (*I* ^2^ = 83.3%; Cochran’s *Q* = 34.7, *p* < 0.001). The funnel plot appears asymmetric, with one study appearing as an outlier^19^ (Figure S8A).

#### 3.7.2 30 ml

Seven studies^15^, ^16^, ^19^, ^20^, ^21^, ^25^, ^26^ had data available to assess maximum diameter measures at 30 ml distention volume. The pooled median was 8.7 mm (5th–95th percentile: 5.6–13.0 mm; 10th–90th percentile: 6.1–11.7 mm). Significant heterogeneity was observed (*I* ^2^ = 87.9%; Cochran’s *Q* = 49.0, *p* < 0.001). The funnel plot appears asymmetric with three outlier studies^16^, ^19^, ^20^ observed (Figure S8B).

#### 3.7.3 40 ml

The pooled median for maximum diameter at 40 ml distention volume was 13.8 mm (5th–95th percentile: 10.2–18.8 mm; 10th–90th percentile: 10.9–17.5 mm), using data from seven studies.^15^, ^16^, ^20^, ^21^, ^22^, ^25^, ^26^ Heterogeneity was present (*I* ^2^ = 90.0%; Cochran’s *Q* = 33.8, *p* < 0.001). The funnel plot appears asymmetric and three studies^20^, ^22^, ^26^ fell outside the triangular 95% confidence region (Figure S8C)

#### 3.7.4 50 ml

Three studies^21^, ^22^, ^26^ had data available on maximum diameter at 50 ml distention. The pooled median was 16.6 mm (5th–95th percentile: 13.5–19.5 mm; 10th–90th percentile: 14.1–18.7 mm). Significant heterogeneity was observed (*I* ^2^ = 90.0%; Cochran’s *Q* = 22.0, *p* < 0.001). Two of the three studies^22^, ^26^ appear outside the triangular 95% confidence region (Figure S8D).

#### 3.7.5 60 ml

Only one study^13^ included data at 60 ml distension volume,^13^ with a median maximum diameter of 18.7 mm (5th–95th percentile: 14.8–23.5 mm; 10th–90th percentile: 15.6–22.4 mm).

## 4 DISCUSSION

An increasing number of studies describe the use of Endoflip™ for evaluation of the EGJ in patients with esophageal symptoms. Differences in median EGJ‐DI between groups of patients with GERD and achalasia and controls have been reported.^5^, ^6^, ^8^ However, without appropriate cut‐off values, it is challenging to draw conclusions on what is considered normal and what is considered pathological. This is why availability of normative values in asymptomatic patients was recently defined as a priority.^2^ There are two methods that can be applied in the search for normal values. One way would be to define definite pathology, for example by describing Endoflip™ measurements in well‐characterized achalasia patients. Another way would be to measure asymptomatic control subjects and define either 5–95th or 10–90th percentiles of these measurements as being normal, values outside this range are then classified as “pathological.” Ideally, the outcome of both approaches provides relatively similar cut‐off values.

In our study, we have extracted values from measurements in healthy, asymptomatic controls from studies published in the literature. Our findings add to the recent review by Desprez et al.^28^ Here, we have pooled all the data and performed a meta‐analysis to describe medians and 5–95th and 10–90th percentiles for EGJ‐DI, intrabag pressure, CSA, and maximal diameter at various inflation volumes. Our findings reflect the values for all measurements published in the literature to date and provides meaningful clinical insight into Endoflip™ measurements in asymptomatic individuals. This manuscript is intended to inform and facilitate proper on‐label use of Endoflip™ technology in symptomatic patients.

The evaluation of whether a patient suffers from achalasia or EGJOO is the most used application of Endoflip™.^5^ For this purpose, the lower cut‐off value for EGJ‐DI is most relevant. At 40 ml fill volume, the lower border of normality is either 1.96 or 2.36 mm^2^/mmHg at 5th or 10th percentile. This is consistent with the use of 2 mm^2^/mmHg as a cut‐off value for definitive abnormality in clinical practice. However, our data reveal that at 40 ml fill volume, 5.4% of healthy subjects have an EGJ‐DI below this value and 20.1% fell below 3 mm^2^/mmHg. Notably, at a fill volume of 50 ml, the percent of measurements for EGJ‐DI below 2 mm^2^/mmHg was only 0.6% in healthy controls and 6.3% of measurements were below 3 mm^2^/mmHg. Further, at a fill volume of 60 ml, the percent of EGJ‐DI measurements below 2 mm^2^/mmHg and 3 mm^2^/mmHg was 0.0% and 7%, respectively. Given these observations, we recommend use of 50 ml for EF‐325N and 50 ml and 60 ml fill volumes for EF‐322N.

In a recent review paper^2,^ an EGJ‐DI between 2 and 3 mm^2^/mmHg was considered indeterminate, but it should be realized that at 40 ml fill volume approximately 20% of healthy subjects will have distensibility below 3 mm^2^/mmHg and in approximately 14% the distensibility will be between 2 and 3 mm^2^/mmHg. It has been recommended to further evaluate the EGJ in these cases by assessing maximal diameter. We found that at 40 ml balloon filling, 5th percentile of maximal diameter was 10.2 and 13.5 mm at 50 ml filling for EF‐325N and 14.8 mm at 60 ml filling for EF‐322N, this means that these values represent the lowest maximal diameter that is still considered normal. Previous recommendations considered a maximum diameter of <13 mm as abnormal,^2^ which seems to correspond to the present values. There are no normal data available for diameter in the situation when the EGJ‐DI is between 2–3 mm^2^/mmHg. However, recent work found that in patients with an EGJ‐DI ≥2.0 mm^2^/mmHg and a maximum EGJ diameter ≥16 mm, 99% had a normal EGJ outflow on high resolution manometry.^29^ The 5th percentile of the intrabag pressure was 20.7 and 31.3 mmHg at the 40 and 50 ml fill volumes, respectively, which is above the recommended pressure of 20 mmHg threshold indicated at 60 ml.^2^

Although there is little discussion about the relevance of an abnormally low EGJ‐DI, it remains the question if it makes sense to define an upper limit of normality for EGJ‐DI. The 95th percentile for upper limit of EGJ‐DI from the meta‐analysis was 10.95 mm^2^/mmHg at 40 ml balloon filling and 10.66 mm^2^/mmHg at 50 ml balloon filling, relatively close to the value of 9 mm^2^/mmHg that has been suggested in the literature.^5^, ^13^, ^17^, ^30^, ^31^ A large spread in values was observed in the different studies. A high EGJ‐DI implies that the EGJ opens easily and predisposes to spontaneous reflux. However, studies show a huge overlap in EGJ‐DI between GERD patients and controls, suggesting the utility of Endoflip™ for GERD diagnosis is limited.^8^ Most likely, this is because GERD is a multifactorial disease, and quantity of reflux is not only determined by a weak EGJ but also by other reflux mechanisms such as transient lower esophageal sphincter relaxations, mucosal barrier function, peristalsis and saliva production and/or reflux sensitivity. On the other hand, subjects may have a very high EGJ‐DI and not suffer from reflux symptoms or esophagitis, it thus seems that distensibility is not suitable for GERD diagnosis. This is why some regard all EGJ‐DI values above 3 mm^2^/mmHg to be normal and question the use of an upper limit for normality of EGJ‐DI.^2^

There are several potential limitations in our study. The data that we have used for the analysis are not extracted from the original individual measurements, as creation of data transfer agreements for all studies would have been required and was deemed prohibitive. Therefore, the meta‐analysis was performed on published values and the spread of the original measurements has been calculated. No sensitivity, subgroups analysis, or meta‐regression were performed due to the small‐scale of the included studies. This could be regarded as a potential weakness, though we felt it was unlikely that all original data from all studies would have been available since some studies were performed over 12 years ago and some authors are no longer active in the field. Therefore, we opted for the most appropriate approach that we could execute in a consistent manner for all studies containing original data.

Furthermore, a large degree of heterogeneity was observed in the studies that were used to extract the data. There were differences in catheters used, age and sex distribution of the subjects, use of sedation and type of sedation, oral versus transnasal intubation, and geographic region. However, when comparing outcome data of studies based on the above‐mentioned factors, reported differences in these factors were unlikely to play a significant role and no trends could be identified. Therefore, we think it was justified to pool these studies for the purposes of our study. Funnel plots created show an asymmetry for several outcomes of interest for this meta‐analysis of normative data for the Endoflip™ system, which could point towards the presence of publication bias, but can also be explained by varied methodological quality, small sample sizes, and/or other bias sources. Finally, a phenomenon called the *dry catheter artefact* was recently described.^32^ The dry catheter artefact is a measurement artifact that impacts diameter measurements when an esophageal contraction at a certain point along the catheter empties pushes away all saline at the point of that contraction and disrupts the electrical current between impedance segments on the catheter, it can result in a temporary erroneously high CSA measurement. The dry catheter artefact is recognized more often nowadays, but it is possible that this was not the case in the earlier studies influenced the data.

In conclusion, using meta‐analysis of studies describing data of Endoflip™ measurements in healthy asymptomatic subjects, we were able to describe the normative range for EGJ‐DI, intrabag pressure, CSA and maximal diameter for various filling volumes. These normative values provide a basis for comparison of values of patients in clinical care and research setting.

## 5 AUTHOR CONTRIBUTIONS

AJB: Study design, data acquisition, results interpretation, manuscript writing, final approval of manuscript. FR: Study design, data acquisition, manuscript writing, final approval of manuscript. HL: Data analysis, manuscript writing, final approval of manuscript. NS: Study conception and design, results interpretation, manuscript writing, final approval of manuscript. MA: Study conception and design, data acquisition, manuscript writing, final approval of manuscript.

## 7 DISCLOSURES

AJB received research funding from Nutricia, Bayer, Norgine, SST and Thelial and received speaker and/or consulting fees from AstraZeneca, Alimentiv, Thelial, Medtronic, Laborie, Regeneron, Sanofi, DrFalkPharma, Reckitt Benckiser, Arena, Calypso.

## 8 DISCLAIMER

This manuscript is intended to inform and facilitate proper on‐label use of Endoflip™ technology in symptomatic patients.

## Supporting information


Figure S1

Figure S2

Figure S3

Figure S4

Figure S5

Figure S6

Figure S7

Figure S8

Table S1
