# Supplementary figures and images for: Normative values for esophageal functional lumen imaging probe measurements: A meta‐analysis
Source: Neurogastroenterol Motil. 2022 Jun 5;34(11):e14419. doi: 10.1111/nmo.14419 (PMC9786273; doi:10.1111/nmo.14419)

### EGJ DI, 20 ML

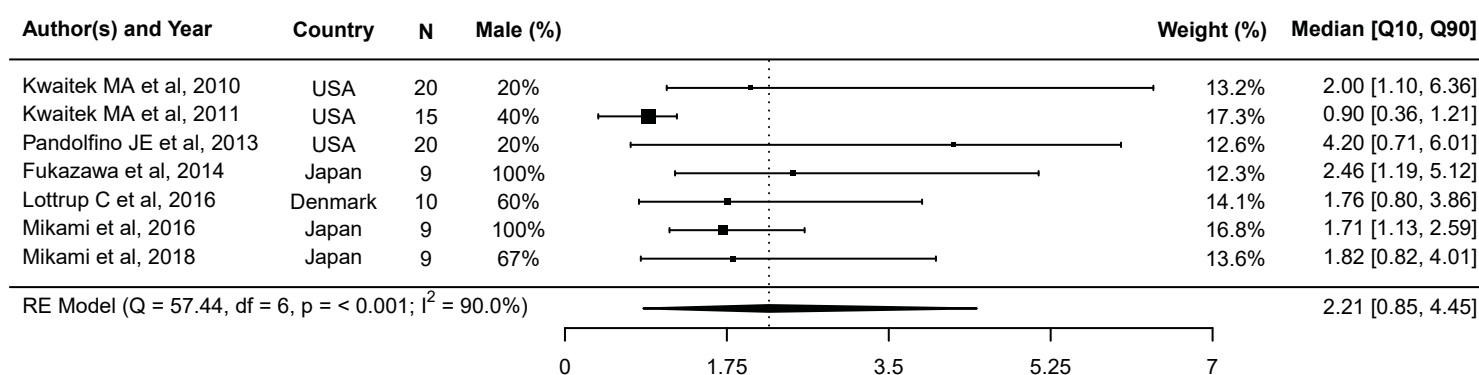

### EGJ DI, 30 ML

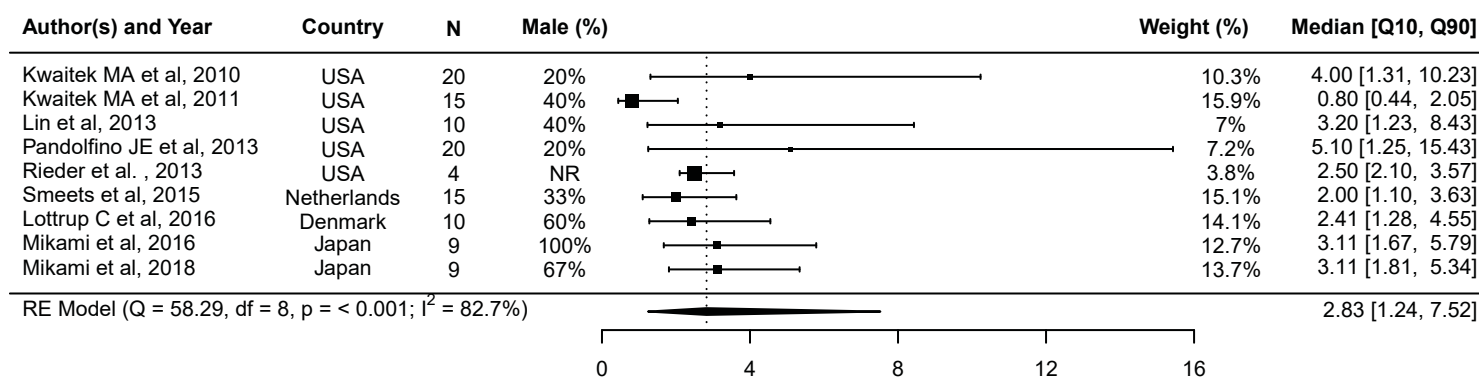

### EGJ DI, 40 ML

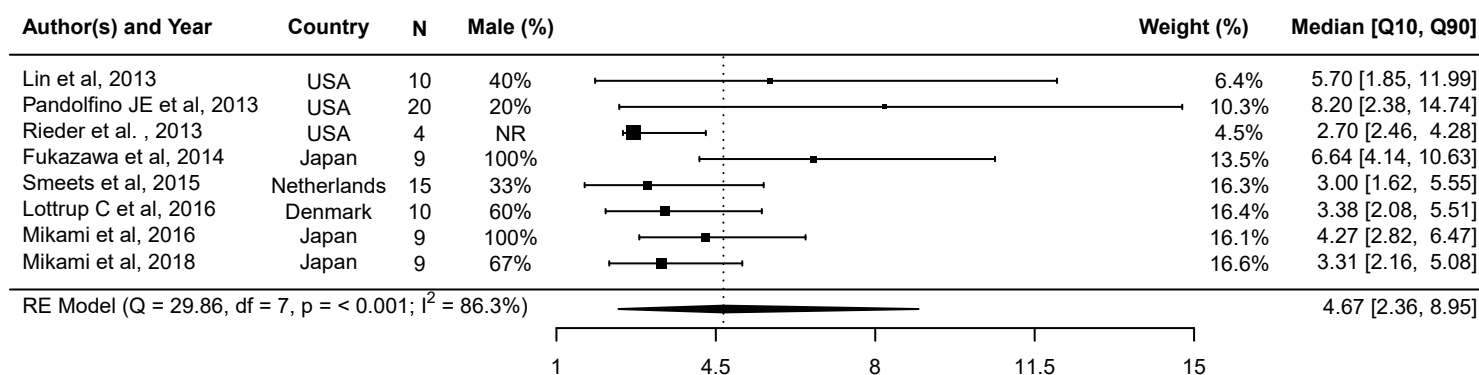

### EGJ DI, 50 ML

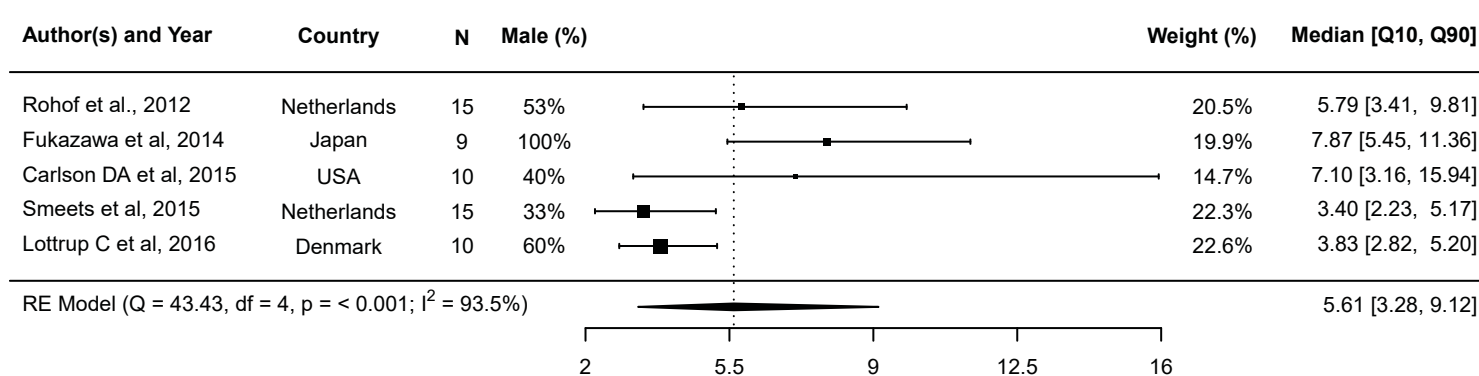

Supplement: Supplementary file 1 — Figure S1 [file NMO-34-e14419-s003.pdf]

### Intrabag Pressure, 20 ML

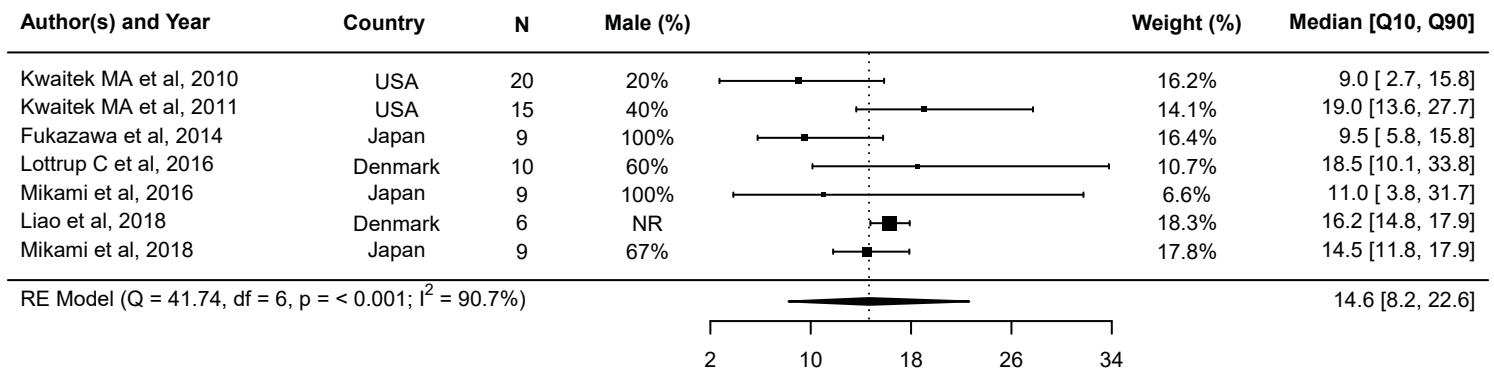

### Intrabag Pressure, 30 ML

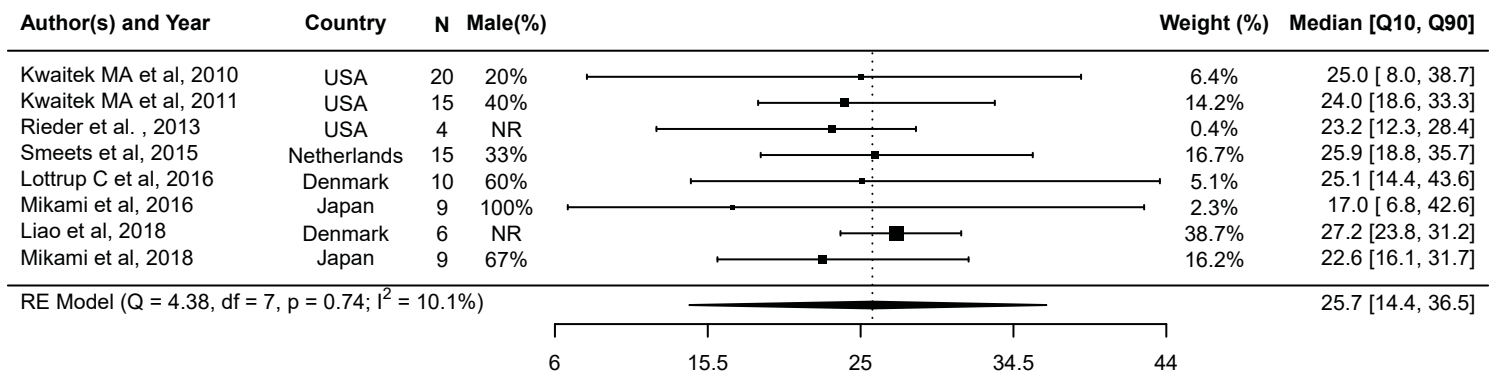

### Intrabag Pressure, 40 ML

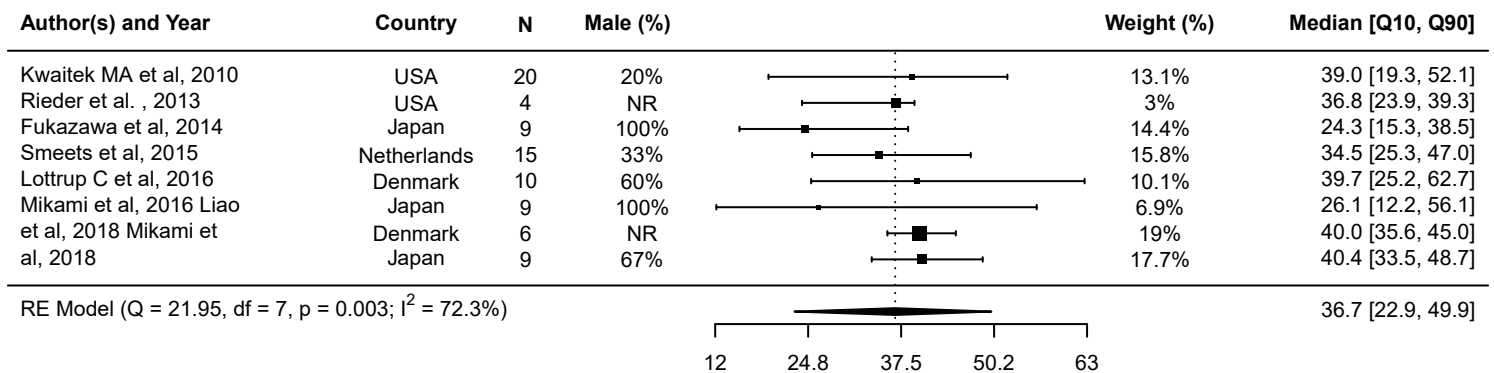

### Intrabag Pressure, 50 ML

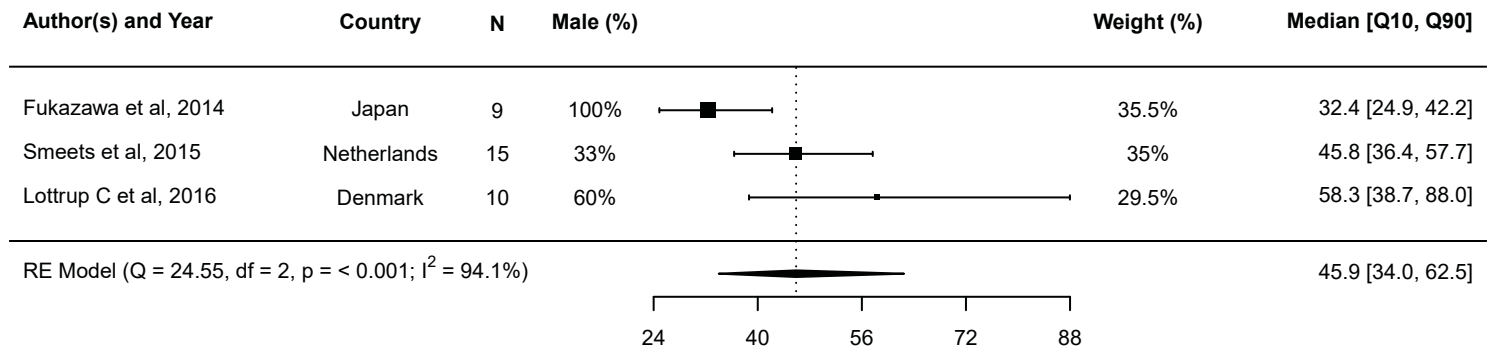

Supplement: Supplementary file 2 — Figure S2 [file NMO-34-e14419-s004.pdf]

### CSA, 20 ML

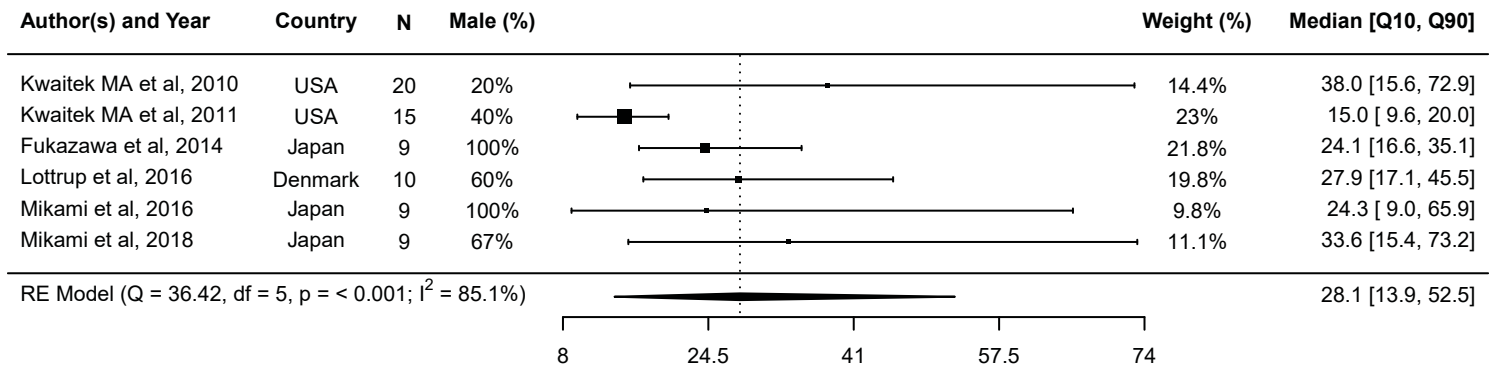

### CSA, 30 ML

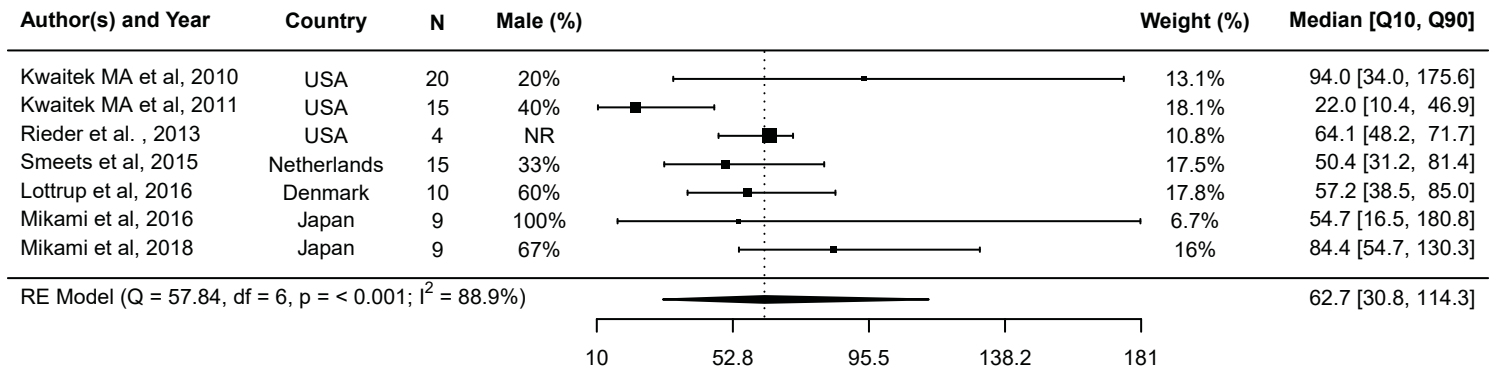

### CSA, 40 ML

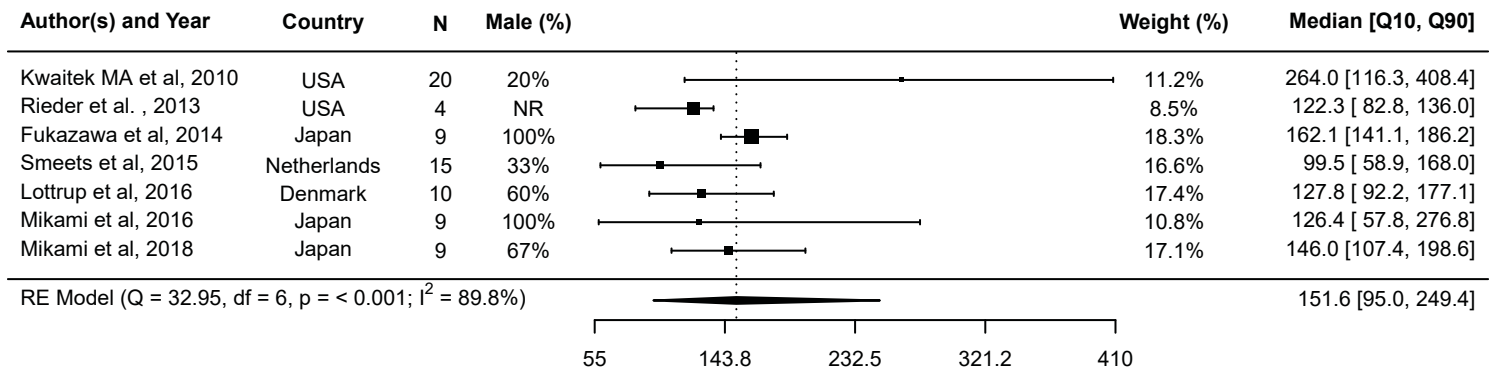

### CSA, 50 ML

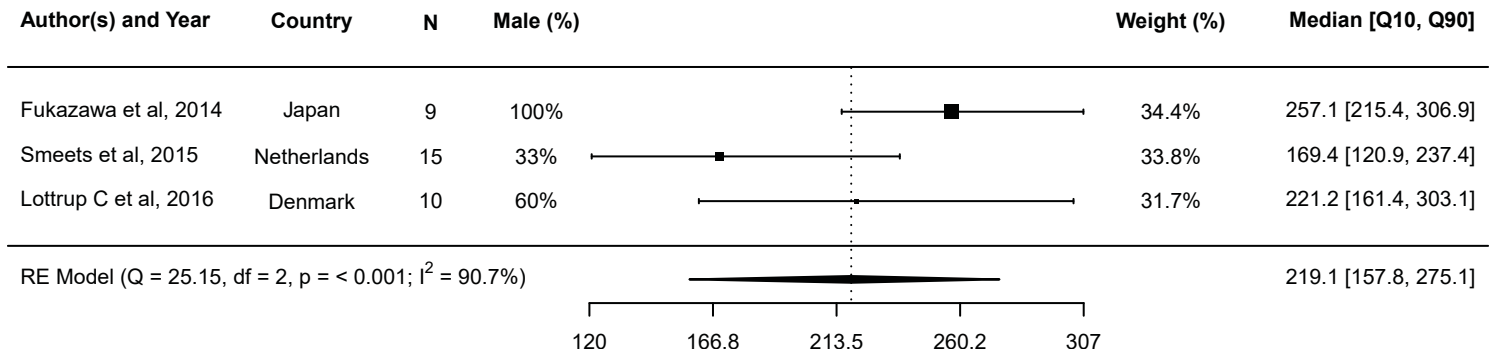

Supplement: Supplementary file 3 — Figure S3 [file NMO-34-e14419-s002.pdf]

### Diameter, 20 ML

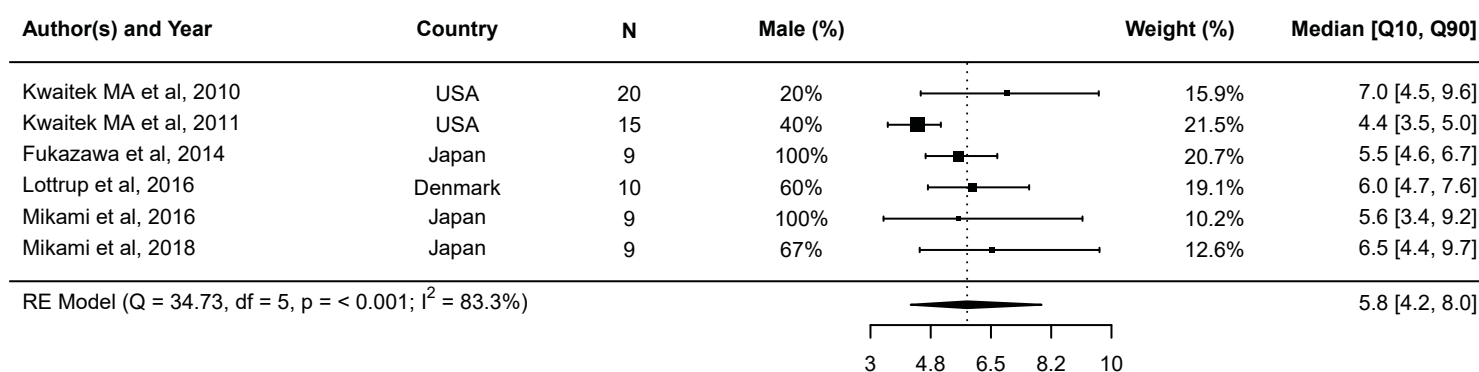

### Diameter, 30 ML

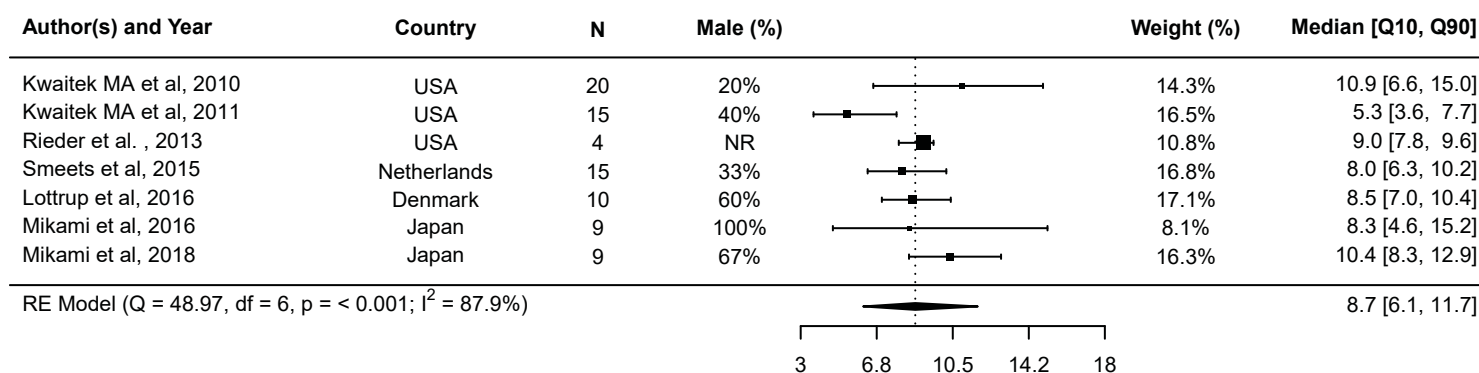

### Diameter, 40 ML

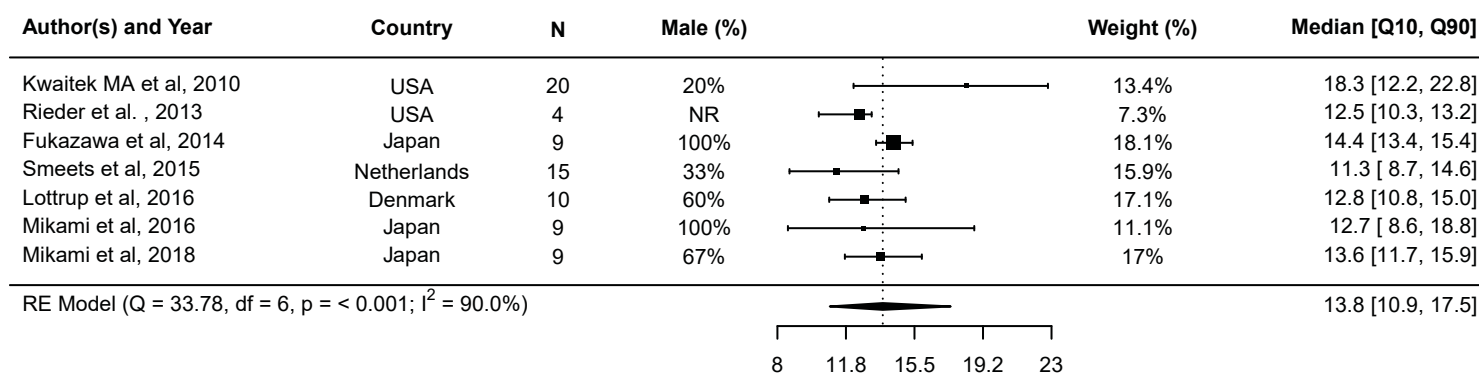

### Diameter, 50 ML

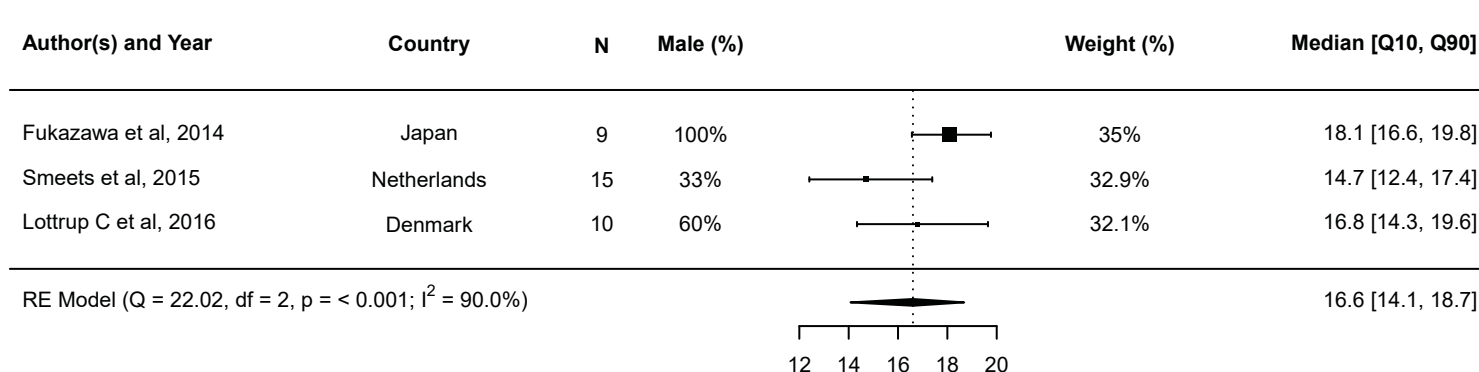

Supplement: Supplementary file 4 — Figure S4 [file NMO-34-e14419-s009.pdf]

### EGJ-DI, 20ML, Funnel Plot

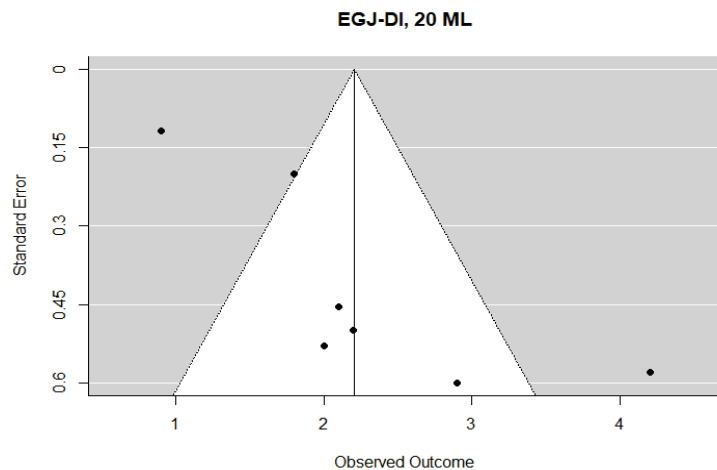

### EGJ-DI, 40ML, Funnel Plot

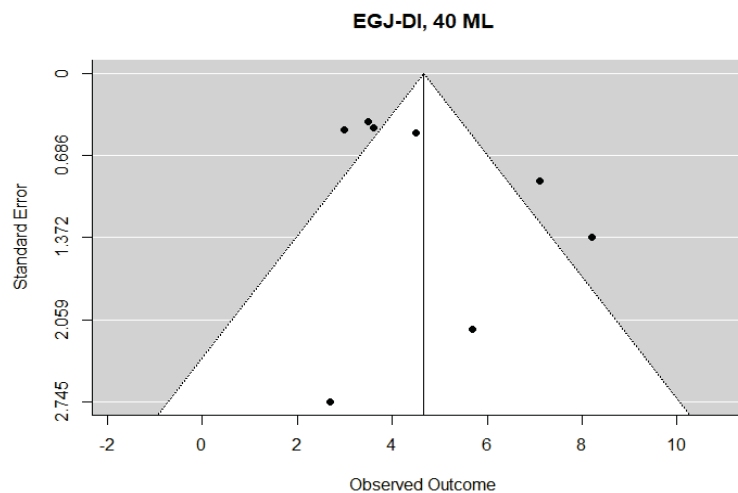

### EGJ-DI, 30ML, Funnel Plot

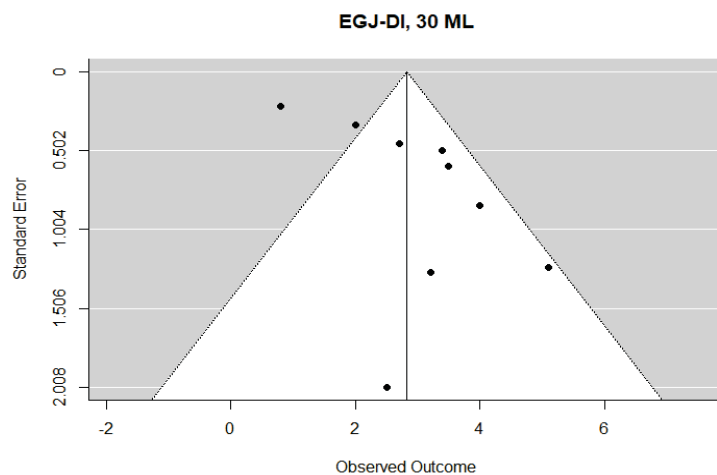

### EGJ-DI, 50ML, Funnel Plot

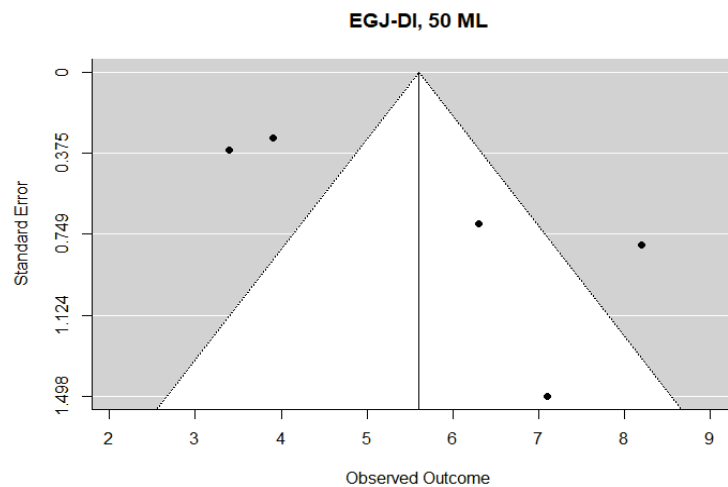

Supplement: Supplementary file 5 — Figure S5 [file NMO-34-e14419-s008.pdf]
