# Supplementary material for: Normative values for esophageal functional lumen imaging probe measurements: A meta‐analysis
Source: Neurogastroenterol Motil. 2022 Jun 5;34(11):e14419. doi: 10.1111/nmo.14419 (PMC9786273; doi:10.1111/nmo.14419)

INTRABAG PRESSURE, 20ML, Funnel Plot

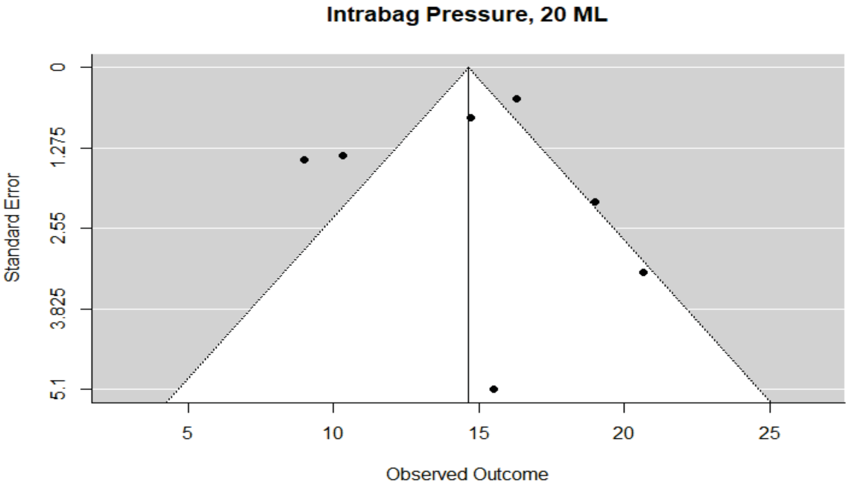

INTRABAG PRESSURE, 40ML, Funnel Plot

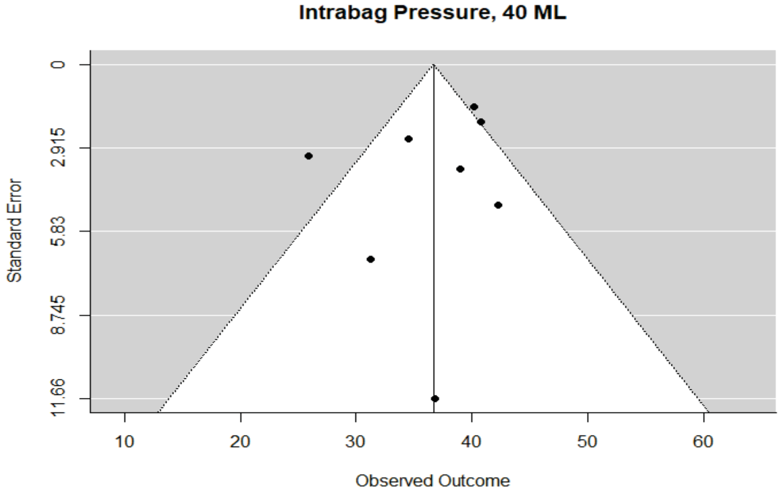

INTRABAG PRESSURE, 30ML, Funnel Plot

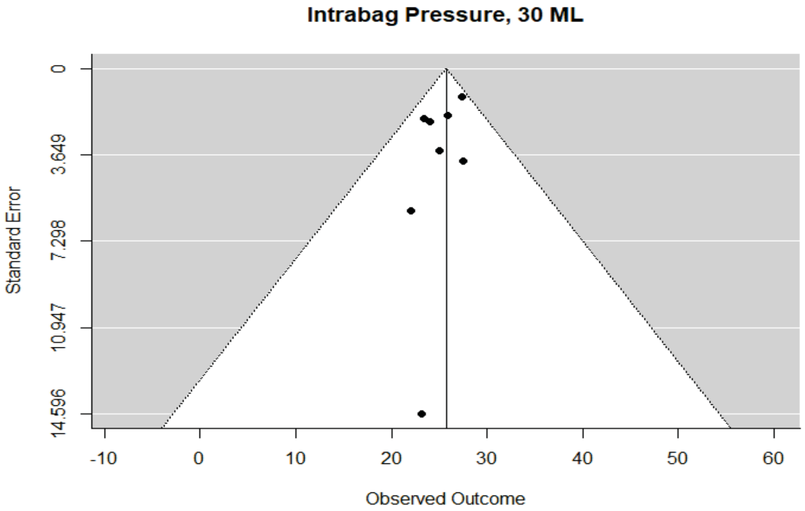

INTRABAG PRESSURE, 50ML, Funnel Plot

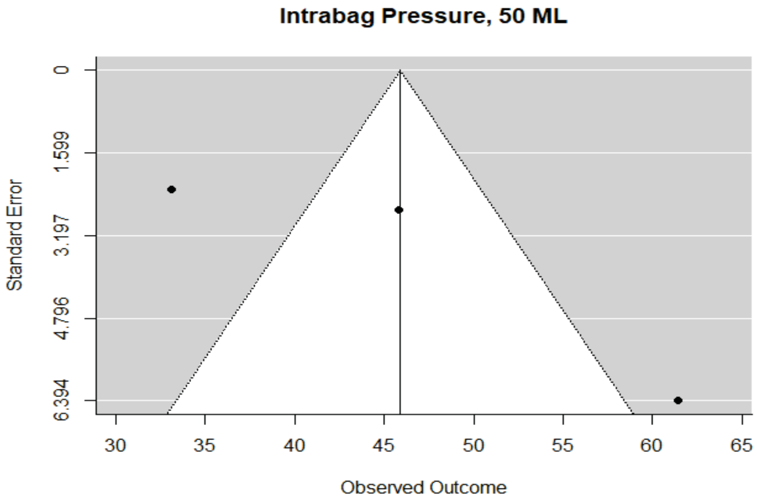

Supplement: Supplementary file 6 — Figure S6 [file NMO-34-e14419-s006.pdf]
