# Supplementary material for: Normative values for esophageal functional lumen imaging probe measurements: A meta‐analysis
Source: Neurogastroenterol Motil. 2022 Jun 5;34(11):e14419. doi: 10.1111/nmo.14419 (PMC9786273; doi:10.1111/nmo.14419)
Supplement: Supplementary file 9 — Table S1 [file NMO-34-e14419-s005.docx]

**Supplementary Table 1. Search Strategy**

**Database(s):** **Journals@Ovid Full Text** December 10, 2019**, Embase** 1974 to 2019 December 10**, Ovid MEDLINE(R) ALL** 1946 to December 10, 2019

| **#** | **Searches** | **Results** |
| --- | --- | --- |
| 1 | (EndoFlip* or Endo Flip*).ti,ab,kw,ct,dm,dv,tn,mf,kf,hw,fx,ec,xt,tx. | 753 |
| 2 | endolum* function* lum* imag* probe*.ti,ab,kw,ct,dm,dv,tn,mf,kf,hw,fx,ec,xt,tx. | 314 |
| 3 | (FLIP* topograph* or (function* lum* imag* probe* and FLIP)).ti,ab,kw,ct,dm,dv,tn,mf,kf,hw,fx,ec,xt,tx. | 846 |
| 4 | ((imped?nce planimet* or (gastric* adj1 tub*)) and Crospon*).ti,ab,kw,ct,dm,dv,tn,mf,kf,hw,fx,ec,xt,tx. | 196 |
| 5 | (abstract* or poster).ti,dt,pt. or (conference* or congress* or meeting* or symposia* or symposium* or (oral* and (presentation* or session*)) or (scientific* and session*) or comment* or editorial* or letter* or note* or patent*).dt,pt. | 23951964 |
| 6 | (1 or 2 or 3 or 4) not 5 | 1105 |
| 7 | limit 6 to yr=2000-current | 704 |
| 8 | english*.lg. or (article in press or "in data review" or in process).st. or aheadofprint.pp. | 131622562 |
| 9 | 7 and 8 | 700 |
| 10 | remove duplicates from 9 | 412 |
